# Study on Long-Term Temperature Variation Characteristics of Concrete Bridge Tower Cracks Based on Deep Learning

**DOI:** 10.3390/s25010207

**Published:** 2025-01-02

**Authors:** Zhaoxu Lv, Youliang Ding, Yan Zhang

**Affiliations:** 1 Jiangsu Xiandai Road & Bridge Co., Ltd., Nanjing 210018, China; 2 Key Laboratory of Concrete and Pre-Stressed Concrete Structures of the Ministry of Education, Southeast University, Nanjing 210096, China; 3 College of Civil Engineering, Southeast University, Nanjing 210096, China

**Keywords:** concrete cable-stayed bridge, LSTM neural network, temperature crack model, temperature field, crack state monitoring

## Abstract

Monitoring existing cracks is a critical component of structural health monitoring in bridges, as temperature fluctuations significantly influence crack development. The study of the Huai’an Bridge indicated that concrete cracks predominantly occur near the central tower, primarily due to temperature variations between the inner and outer surfaces. This research aims to develop a deep learning model utilizing Long Short-Term Memory (LSTM) neural networks to predict crack depth based on the thermal variations experienced by the main tower. The efficacy of the LSTM network will be rigorously evaluated, employing multiple temperature input datasets to account for spatial dimensional variations in the data. This methodology is anticipated to enhance the model’s accuracy in predicting crack widths. By leveraging the deep learning regression model, precise temperature thresholds for crack formation can be established, facilitating early detection of anomalies in the crack widths of the main tower and providing effective technical solutions for monitoring crack status.

## 1. Introduction

Concrete cable-stayed bridges are subjected to a complex interplay of internal and external factors throughout their service life, including traffic growth, the presence of overloaded vehicles, and temperature fluctuations. These conditions contribute to an increasing prevalence of structural issues such as cracks, permanent deformations, and geometric defects, with cracks emerging as the most common and severe form of deterioration observed in concrete cable-stayed bridges [1]. Existing research has substantiated that these cracks are predominantly concentrated around the central tower columns. Consequently, elucidating the mechanisms underlying crack development in cable-stayed towers and conducting comprehensive safety assessments of cracked cable-stayed towers have become paramount for ensuring structural integrity and safety [2,3].

Bridges are subjected to a diverse array of loads throughout their operational lifespan. Among these loads, wind and vehicle loads are classified as direct effects, while foundation deformation, material creep and shrinkage, along with temperature variations, fall under the category of indirect effects [4]. Extensive research and practical observations have established that temperature fluctuations play a significant role in the initiation and progression of cracks within the components of concrete cable-stayed bridges [5]. During the processes of casting, hardening, and subsequent service, concrete experiences substantial thermal stress attributable to hydration heat and variations in external air temperature. These factors contribute to alterations in the internal temperature gradient of the concrete, thereby inducing non-negligible thermal expansion and contraction effects [6]. If such temperature changes are not effectively managed, they can markedly increase the risk of cracking, particularly in the cable towers of cable-stayed bridges. Over time, these initial cracks may propagate, posing serious challenges to the long-term structural integrity of the bridge [7]. Therefore, it is imperative to conduct comprehensive research on the characteristics of temperature variations and the long-term evolution of existing cracks in cable-stayed concrete bridge towers. Such investigations are essential for improving bridge maintenance management practices and ensuring the safety and reliability of bridges over their entire lifecycle.

Extensive research has been conducted on the monitoring of cracks in cable-stayed bridges. For example, Xia Min et al. [8] investigated a cable-stayed bridge and determined that the cracks observed in the web of the lower crossbeam of the bridge tower were primarily attributable to factors such as concrete shrinkage, temperature differentials between the interior and exterior of the chamber, and variations in hardening temperatures. Their findings were based on monitoring force conditions during construction along with finite element analysis. Similarly, Duan et al. [9] developed a finite element model of the main tower to analyze the influence of the temperature field on its structural integrity, establishing parameters necessary for calculating the temperature distributions. Their study specifically examined the impact of solar radiation on the temperature field of the main tower over a 24 h period. Additionally, Xu Feng et al. [10] focused on the H-type concrete bridge tower of the Taizhou Bay Bridge, employing three-dimensional heat transfer simulation and analysis software while integrating measured meteorological data to construct a comprehensive temperature field model. Their analysis encompassed changes in internal and external surface temperatures, characteristics of radial temperature distribution, and the implications of temperature variations on tower displacement and stress distribution. In summary, a multitude of researchers have performed extensive investigations into the distribution of temperature fields and thermal effects within concrete box girders and main towers, yielding valuable insights that form a robust foundation for further inquiry in this domain [11]. Nevertheless, there remains a notable gap in the investigation of temperature effects on existing cracks in concrete bridge towers, particularly concerning the cracking mechanisms specific to cable-stayed towers and the long-term evolution of these cracks.

With the development of intelligent algorithms, prediction algorithms based on deep learning have been significantly advanced, which contributes to the comprehensive application of bridge structures [12,13]. By establishing a time-series prediction model for concrete cracks in bridges, real-time crack monitoring and reduction in maintenance costs can be effectively improved. Relevant studies have shown that models such as BPNN [14], LSTM [15], Gated Recurrent Units (GRUs) [16], and transformer-based [17] have been applied in crack predictions and others. However, among them, the LSTM model is distinguished by its incorporation of distinctive memory cells and an intricate gating mechanism, which can independently modulate the storage and discarding of information during the neural network training, discarding irrelevant historical data and preserving past information that significantly influences the output results. Thus, the gradient vanishing issue stemming from excessive historical information is effectively mitigated, and the network’s capacity to model long-term dependencies is enhanced [18,19].

This paper focuses on the Huai’an Bridge as the research subject. It utilizes measured data on crack width and tower temperature to investigate the characteristics of temperature variation in relation to the crack width of the cable tower, employing health monitoring data and a deep learning approach. By implementing a high and low-frequency hierarchical learning method, along with comprehensive temperature field analysis of the entire tower, an intelligent real-time high-precision prediction model for crack width is developed. Additionally, the residual difference between the predicted and measured values is utilized to characterize abnormal response information, enabling direct assessment of specific abnormal crack width values. The sensitivity of this abnormal detection can be enhanced by compressing the sensing frequency, providing valuable insights for the prevention and control of further crack propagation.

## 2. Huai’an Bridge Tower Monitoring Data Analysis

### 2.1. Temperature Monitoring Data

#### 2.1.1. Bridge Monitoring Deployment and Data Collection

The Huai’an Bridge spans a total length of 2062 m and features a configuration of six lanes in each direction. The main bridge section is designed as a prestressed concrete double-tower, double-surface cable-stayed bridge with a fully floating system, incorporating a span layout of 152 m + 370 m + 152 m. The towers are constructed using a reinforced concrete box structure and were erected through an overturning process, reaching a height of 137.1 m, with two layers of beams positioned between them. Each tower leg is equipped with 31 pairs of cable-stayed cables, resulting in a total of 248 cables for the bridge; the number of steel strands in individual cables varies from 34 to 61. The towers exhibit an “H”-type structure, with upper and middle columns measuring 77 m and 47 m in height, respectively. Given that each tower leg of the Huai’an Bridge receives similar solar radiation, only the upstream tower leg on the Huai’an side has been selected as the primary measurement point for monitoring tower temperature.

**Figure 1:**
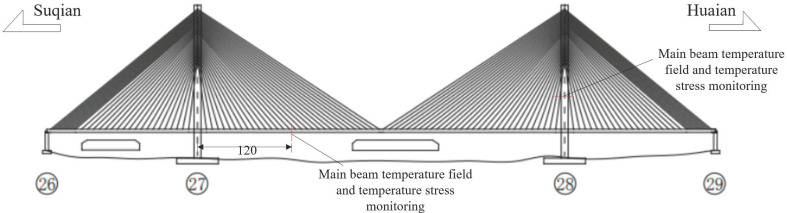
Monitoring cross-section arrangement of Huai’an Bridge (unit: m).

The internal measurement point is located at a height of 30 m within the tower, while external measurement points are positioned near the main tower and girder. Each surface of the tower wall, both inside and outside, features measurement points oriented toward the southeast, northwest, and north (for a total of eight measurement points). The layout of the monitoring sections of the bridge tower, along with the configuration of the temperature measurement points across the cross-section, is illustrated in Figure 1.

**Figure 2:**
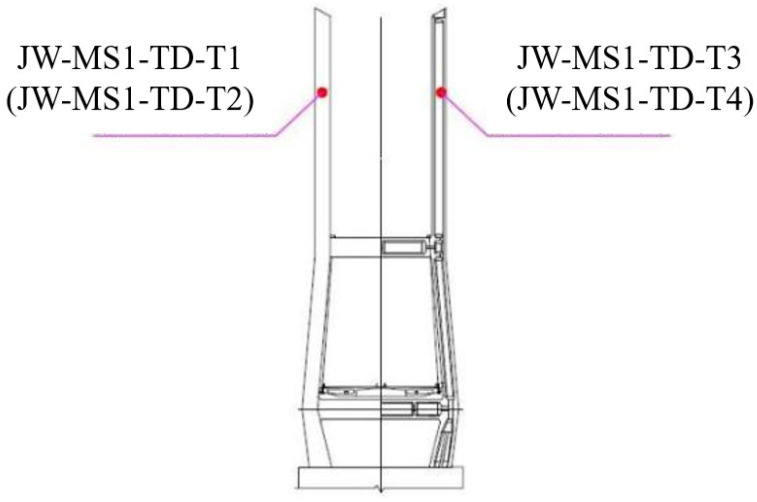
Arrangement of temperature measurement points of the main tower on the Suqian side.

Figure 2 illustrates the layout of the temperature measurement points on the main tower at the Suqian side. The temperature sensors used are vibrating string strain gauges with a measurement error of ≤2, which collect data every five minutes. Sensors JW-MS1-TD-T1 (referred to as JW-T1) and JW-T2 are positioned in the upstream region of the main tower’s cable anchorage, specifically in the longest and intermediate cable anchorage areas. Conversely, JW-T3 and JW-T4 are located downstream within the same anchorage zones. All temperature sensors are mounted externally on the main tower.

Data acquisition may experience anomalies due to inherent equipment defects, environmental influences, unstable transmission signals, or other factors, leading to unexpected data irregularities. The most common types of anomalies observed include data jumps and data drift [20]. There are some jump points in the temperature monitoring data in this paper, and the generalized 3-sigma algorithm is chosen in this paper for comprehensive consideration. Based on the 3-sigma rule (i.e., the probability that values falling within 3 standard deviations of the mean are concentrated near the mean is close to 99.7%) to clean the jump points, it has a wide range of applications and effectively cleans the jump points in the data of this study. To analyze the temperature variation characteristics relevant to crack width, it is essential to focus on the main trends in the temperature data. Accordingly, this Section utilizes the wavelet packet decomposition method to process the temperature data, reducing computational burdens while maintaining accuracy.

#### 2.1.2. Temperature Data Analysis Based on Wavelet Packet Decomposition

**Figure 3:**
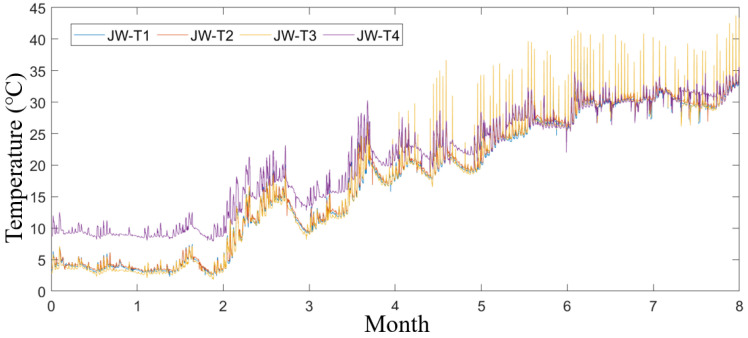
Data after wavelet packet decomposition for each temperature sensor data in 2022.

The wavelet packet decomposition technique is widely utilized in signal analysis, demonstrating superior performance in resolving high-frequency components compared to traditional wavelet analysis [21]. By applying wavelet packet decomposition to the original data from each temperature sensor, the resulting temperature data are presented in Figure 3. From January to August, the temperatures measured by each sensor exhibited a gradual increase, with similar trends observed across all sensors. Notably, the temperature recorded by JW-T4 during the first five months was relatively high, while the remaining sensors reported values that were closely aligned with one another. Nevertheless, considering the results of the actual field measurements and the environmental impacts, the trend and distribution of the changes are consistent, and these data are suitable for conducting subsequent studies.

### 2.2. Crack Width Monitoring Data

#### 2.2.1. Data Collection

Based on the results of field tests, four cracks with distinct characteristics were selected as the focal subjects of this study. These cracks are situated on the downstream side wall of the tower’s top, the outer roof plate of the middle transverse tie beam, the junction between the middle tower column and the middle transverse beam, and the junction between the middle tower column and the lower transverse beam. Correspondingly, crack sensors LF-12-01 through LF-12-04 were installed at these locations, with all sensors configured to collect data at a frequency of once every five minutes. The crack monitoring sensors employed in this study are thin-film crack meters, which feature a measurement accuracy of ±1 and an operating temperature range of −50 °C to 150 °C. Importantly, several jump points were identified within the crack data; therefore, data cleaning was performed using the same methodology applied to process the temperature data.

#### 2.2.2. Crack Width Data

**Figure 4:**
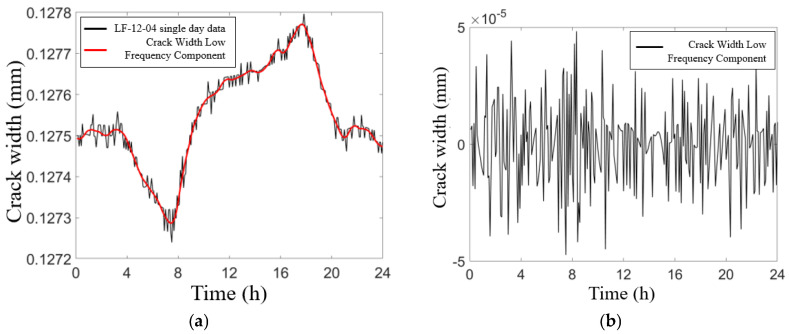
Wavelet packet decomposition of sensor LF-12-04 single-day data: (**a**) Crack width raw data and low-frequency component. (**b**) Crack width HF component.

**Figure 5:**
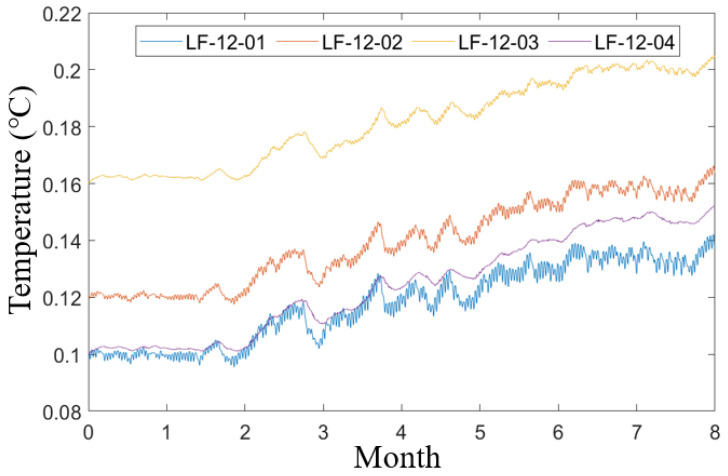
Data after wavelet packet decomposition of each crack width sensor data.

The local fluctuations observed in the crack data are predominantly high-frequency components induced by vehicle loading and various other factors, while the global fluctuations are characterized as low-frequency components that arise primarily from temperature effects. To extract the principal components from the data collected by the crack sensors, the wavelet packet decomposition method [21] was employed. Figure 4 illustrates the wavelet packet decomposition of the data recorded by sensor LF-12-04 on May 21. Specifically, Figure 4b highlights the high-frequency components of crack width attributable to vehicle loading and other influences. Furthermore, Figure 5 presents the processed crack width data from each sensor. The measured trends for all cracks exhibit a degree of uniformity, with crack widths gradually increasing from January to August, ultimately reaching their maximum values during this period in August. The maximum crack widths recorded by sensors LF-12-01 through LF-12-04 were 0.142 mm, 0.166 mm, 0.205 mm, and 0.152 mm, respectively.

#### 2.2.3. Analysis of the Correlation Between Crack Width and Temperature

**Figure 6:**
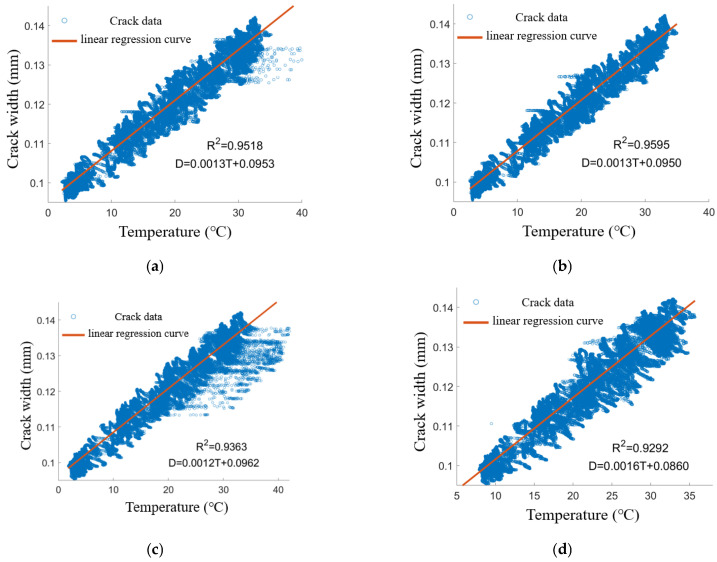
Correlation scatter plot of temperature characteristics with crack width data and one-way linear regression analysis: (**a**) JW-T1 and LF-12-01. (**b**) JW-T1 and LF-12-02. (**c**) JW-T1 and LF-12-03. (**d**) JW-T1 and LF-12-04.

The temperature characteristics of dataset JW-T1 were plotted against the crack widths from sensors LF-12-01 to LF-12-04 (denoted as *D*) using a scatter plot and one-way linear regression analysis, as depicted in Figure 6. The regression value for crack width was recorded as *D*’, with the fitting results assessed using the goodness-of-fit indicator (*R*2). While a clear linear correlation exists between the crack widths measured from JW-T1 and the temperature characteristics captured by the sensors at various locations, there is also a notable degree of nonlinearity and dispersion present in the data. This complexity poses challenges for achieving satisfactory results through one-dimensional linear regression analysis. Although the most favorable outcomes were obtained using the temperature data from the same-side tower limb LF-12-02 (with *R*2 = 0.9595), the correlation plot still exhibited several outliers. These outliers can be attributed to the intricate spatial and temporal distribution of the temperature field, as well as the complex interplay between crack width and temperature characteristics.

The linear regression equation derived from the temperature data at measurement point JW-T1 to model the thermotropic crack width at measurement point LF-12-02 is expressed as (*D* = 0.0013 T + 0.0950). This equation can also be utilized to predict the crack width at LF-12-02 based on the temperature data from JW-T1. It was observed that the predicted crack width data for measurement point LF-12-02 closely align with the overall trend of the actual values; however, a significant discrepancy between the predicted and actual values remains apparent. This suggests that relying solely on the goodness-of-fit indicator (*R*2) for assessing prediction accuracy may not be sufficient. Therefore, to enhance automated detection of anomalous crack widths and improve the real-time accuracy of the evaluation model, the Absolute Error (*AE*) for each data point will be analyzed. The *AE* can be calculated using Equation (1):

(1) $$AE={D^{\prime }}_{n}-D_{n}$$

where *D_n_*’ is the predicted value of crack width, and *D_n_* is the true value of crack width.

Furthermore, this paper introduces a definition of prediction accuracy (*P*), which specifically includes the mean absolute error (MAE, denoted as (*ρ_R_*)) between the predicted values and the corresponding true values of the principal components. Additionally, it incorporates the mean absolute error after removing the main trend; this involves simultaneously decomposing both the predicted and true values using wavelet packet analysis to extract the high-frequency components before calculating the MAE (denoted as (*ρ_r_*)). This value is then normalized by the mean of the corresponding true values of the target data. Given the significant proportion of the principal components, the prediction accuracy (*P*) is defined as shown in Equation (2):

(2) $$P=0.7\rho_{R}+0.3\rho_{r}$$

*ρ_R_* and *ρ_r_* are solved as in Equations (3) and (4):

(3) $$\rho_{R}=\left(1-\left(\frac{1}{n}\sum_{i=1}^{n}\left|\left|Y_{i}\right|-\left|{Y^{\prime }}_{i}\right|\right|\right)/\left(\frac{1}{n}\sum_{i=1}^{n}\left|{Y^{\prime }}_{i}\right|\right)\right)\times 100\%$$

where *Y_i_* and *Y_i_*’ represent the true and predicted values of the principal components at *i*, respectively, and *n* is the total number of predicted data.

(4) $$\rho_{r}=\left(1-\left(\frac{1}{n}\sum_{i=1}^{n}\left|\left|y_{i}\right|-\left|{y^{\prime }}_{i}\right|\right|\right)/\left(\frac{1}{n}\sum_{i=1}^{n}\left|{y^{\prime }}_{i}\right|\right)\right)\times 100\%$$

where *y_i_* and *y_i_*’ represent the true and predicted values after detrending at *i*, respectively, and *n* is the total number of predicted data.

**Figure 7:**
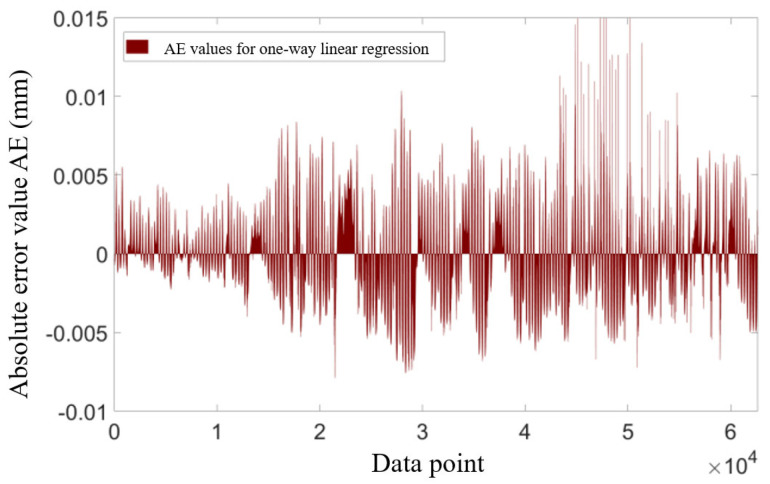
Absolute error in the prediction of the one-way linear regression equation for crack width at the JW-T1 and LF-12-02 measurement points AE.

Figure 7 illustrates the AE for each data point in the test set. It is observed that the mean value of AE is close to zero; however, the output accuracy remains unstable, particularly in the latter half of the dataset, where AE shows significantly larger values. The prediction accuracy (*P*), derived from linear regression and calculated using the definition provided in Equation (2), is only 76.23%. This indicates that the data-driven model established through one-dimensional linear regression does not meet practical requirements. A similar one-dimensional linear regression analysis was performed for the remaining three cracks using temperature characteristics from sensors at various locations, revealing conditions analogous to those observed with JW-T1.

## 3. Intelligent Real-Time Prediction Modeling of Crack Widths in Sotahs Bridge Tower

### 3.1. LSTM-Based Real-Time Prediction Model

#### 3.1.1. LSTM Model

Deep learning is fundamentally rooted in the study of artificial neural networks, which emulate the interactions and learning processes among neurons in the human brain by constructing multilayer nonlinear models [22]. Among these various architectures, the Backpropagation Neural Network (BPNN) serves as a foundational framework for many deep neural network architectures. The Long Short-Term Memory Network (LSTM), an advanced architecture of Recurrent Neural Networks (RNNs) [23], has been specifically designed to address the long-term dependency issues encountered by conventional RNNs during the training process. This capability makes LSTMs particularly effective in tasks that require the retention of information over extended periods, such as time-series prediction, natural-language processing, and various applications within the civil engineering domain where monitoring and predicting structural cracks over time is essential.

**Figure 8:**
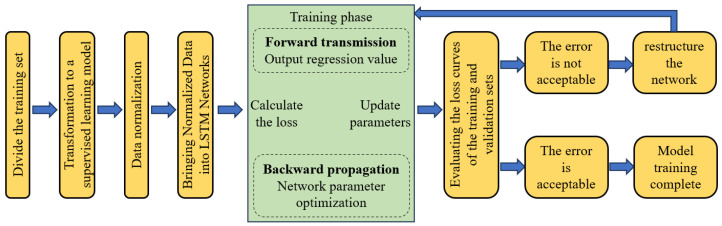
Flowchart of training model for real-time prediction of bridge tower crack width by LSTM neural network.

Figure 8 provides a comprehensive flowchart that outlines the training process for the real-time prediction model aimed at estimating the crack width of bridge towers utilizing an LSTM neural network. Initially, the dataset is transformed into a supervised learning format after the construction of both training and testing sets. Concurrently, the temperature feature data and crack width measurements are normalized to ensure consistency and improve model performance. Furthermore, this study employs the Adam optimization algorithm as the gradient descent optimizer, which is specifically designed to enhance convergence efficiency in training deep learning models. In the context of regression analysis, the mean squared error (MSE) between the predicted values and the actual outcomes is typically utilized as the loss metric. This loss metric is mathematically defined by the following formula:

(5) $$loss=\frac{1}{N}{\sum_{n=1}^{N}\left(d_{n}-{d^{\prime }}_{n}\right)}^{2}$$

where *d_n_*’ is the regression value before inverse normalization; *d_n_* is the normalized actual value of the thermotropic response; and *N* is the number of data points.

Following the preprocessing of data within the training set, the iterative training process is initiated. This phase necessitates a predetermined number of iterations, commonly referred to as epochs. Upon reaching this specified iteration count, the training phase is officially concluded. If the error curves, also known as loss curves, for both the training and validation datasets demonstrate a converging trend, it signifies that the model’s error levels and regression performance are satisfactory, thereby indicating the successful completion of the training phase. Importantly, the processes of data preprocessing and forward propagation during the inference stage mirror those utilized in the training phase. Initially, the network generates the normalized regression output (denoted as (*d*’)). As illustrated in Equation (6), the regression value (denoted as (*D*’)) corresponding to the thermotropic crack width can be obtained by back-normalizing the normalized output ((*d*’)).

(6) $$D^{\prime }=\left(D_{\max}-D_{\min}\right)d^{\prime }+D_{\min}$$

where *D*_max_ and *D*_min_ are the maximum and minimum values in this thermotropic response variable, respectively.

To attain optimal fitting, crack width and temperature data sourced from the pylons of the Huai’an Bridge, collected between January and August 2022, underwent a thorough data cleaning process, which included the removal of NaN (Not a Number) values. This preprocessing step resulted in a total of 62,662 data points for each temperature feature variable, along with the corresponding crack width measurements. The initial 75% of the dataset, encompassing 46,996 data points, was designated as the training set, while the remaining 25%, consisting of 15,666 data points, was allocated as the test set.

#### 3.1.2. LSTM-Based Numerical Regression of Thermotropic Crack Widths

In this study, only the temperature data collected from the tower limb on the same side were utilized as the temperature feature dataset for input into the LSTM model. Given that all four cracks are situated downstream, JW-T1 was selected to serve as the temperature feature dataset for LF-12-01 and LF-12-02. Similarly, JW-T2 was designated as the temperature feature dataset for LF-12-03 and LF-12-04, in accordance with the spatial distribution of the cracks.

Numerical regression using LSTM neural networks necessitates converting the dataset into a supervised learning mapping model. Previous studies have indicated that incorporating data from the first 5 h prior to the current moment yields better results concerning the effects of temperature. Thus, data from each temperature sensor within the initial 5 h (specifically, the first 60 data points) are utilized as input. Enumeration begins from the 30th moment, as detailed in Table 1, where T1 and T2 represent the JW-T1 and JW-T2 datasets, respectively. In contrast to the LSTM network, the BP (Backpropagation) network does not require the decomposition of temperature feature data into separate inputs corresponding to different moments; instead, a uniform input to the network is sufficient.

In this study, the Deep Learning Toolbox of the MATLAB platform was employed to construct the neural network. The computer’s specifications included a Core i7-7700K CPU operating at 4.2 GHz, along with 16 GB of RAM. For the neural network hyperparameters, the minibatch size was set to 32, and the initial learning rate was established at 0.0001. The LSTM input layer was configured with a total of 60 units, while the input size was set to 1. The training set data were fed into the LSTM model, and the LSTM parameters were continuously optimized during backpropagation using Adam’s algorithm. After a designated number of epochs, a trained LSTM model for predicting temperature-induced crack width was successfully obtained.

**Figure 9:**
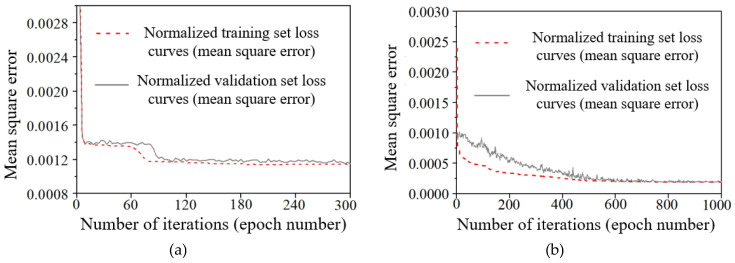
Loss curves of the normalized dataset for the training process of BPNN network and LSTM network: (**a**) BPNN-5 hidden layers-128 units per layer. (**b**) LSTM-2 hidden layers-128 units per layer.

Through a comprehensive series of experimental tests, it was found that the goodness-of-fit (*R*2) and prediction accuracy (*P*) of the test set exhibited only marginal improvements when the number of hidden layers in the Backpropagation (BP) neural network was configured to five, with each layer containing 128 hidden units. Under these conditions, the prediction accuracy (*P*) for the test set stabilized at approximately 78%. In a similar vein, when the Long Short-Term Memory (LSTM) network was designed with two hidden layers, each comprising 128 hidden units, its goodness-of-fit (*R*2) and prediction accuracy (*P*) demonstrated negligible increases, with P approaching 80%. The training and validation loss curves for both the established BP and LSTM models are illustrated in Figure 9. It is clear that the BP model converges after fewer iterations compared to the LSTM model; however, this faster convergence is accompanied by larger error margins. Overall, the fitting performance of the LSTM network significantly surpasses that of the BP network, as well as the outcomes obtained from univariate linear regression.

Furthermore, the Long Short-Term Memory (LSTM) network exhibits a marked improvement in goodness-of-fit (*R*2) scores. In addition to this enhancement in *R*2, the LSTM’s capability for representing nonlinear relationships in the time domain significantly mitigates the time lag phase error associated with the fitted values. As a result, the prediction accuracy (*P*) of the regression values derived from the LSTM network surpasses that of the Backpropagation (BP) network by 2%. This difference is particularly pronounced in scenarios where there is a strong low-frequency correlation (*ρ_R_*), indicating enhanced performance in capturing the high-frequency components (*ρ_R_*). The robust accuracy of both amplitude and phase in the outputs generated by the LSTM network further improves the alignment between the model’s regression values and the actual observed values, thereby enhancing overall predictive capabilities when compared to the BP network.

### 3.2. High–Low-Frequency Layered Learning Approach

**Figure 10:**
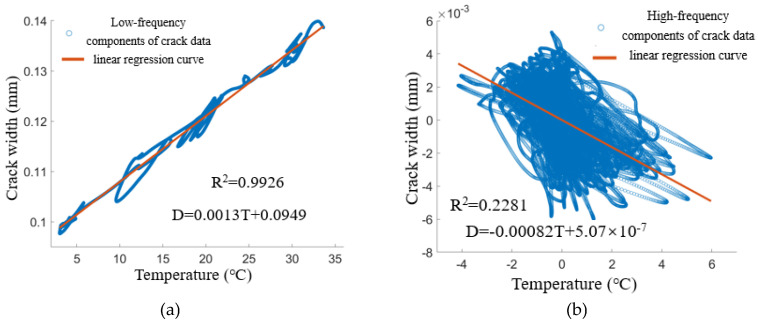
Correlation analysis of high and low frequency components of temperature data and crack width data: (**a**) Low-frequency component correlation. (**b**) High-frequency component correlation.

The one-dimensional linear regression and Long Short-Term Memory (LSTM) models previously discussed capture only the general trend between temperature and crack width, failing to effectively address the specific details of their relationship. To overcome this limitation, this study introduces a novel learning model based on a hierarchical learning approach that differentiates between high-frequency and low-frequency components. This methodology involves first extracting temperature and crack width data simultaneously through wavelet packet decomposition, enabling the identification of both high-frequency and low-frequency components. Following this, the high-frequency and low-frequency data are modeled separately and subsequently combined to generate comprehensive predicted values. The crack dataset LF-12-01 and the temperature dataset JW-T1 are utilized as case studies to assess the feasibility of this hierarchical learning method. Figure 10 illustrates the correlation analysis of the decomposed high-frequency and low-frequency temperature data in conjunction with crack width data. The low-frequency components exhibit a very strong positive correlation, evidenced by a goodness-of-fit (*R*2) value of 0.9926. In contrast, the high-frequency components display a certain degree of negative correlation, with a goodness-of-fit (*R*2) value of 0.2281.

The LSTM neural network models for both the high-frequency and low-frequency components were trained following the established algorithmic process. The input parameters for these models were configured as follows: the minibatch size was set to 32, the initial learning rate to 0.0001, the total number of units in the LSTM input layer to 60, the input size to 1, and both the number of hidden layers and the number of hidden units were set to 2 and 128, respectively. Table 2 presents a comparison of the results from the crack dataset LF-12-01 and the temperature dataset JW-T1 under these identical parameter settings. It contrasts the outcomes from directly applying the LSTM network for deep learning with those obtained through the hierarchical learning method that differentiates between high and low-frequency components. Both methodologies exhibit relatively high goodness-of-fit values, approaching 1. However, the prediction accuracy achieved using the hierarchical learning method is 87.11%, significantly higher than the 79.58% attained through the direct application method. This indicates that the high and low-frequency hierarchical learning approach substantially enhances prediction accuracy.

In this Section, the optimal input parameters for the LSTM network and the input methods for the datasets were thoroughly examined. Consequently, LSTM neural network models were established using the high and low-frequency hierarchical learning method for each of the crack datasets associated with sensors LF-12-01 to LF-12-04. Table 3 presents the computational accuracy of the LSTM model for each sensor’s test set data. As shown in Table 3, compared to univariate linear regression, the goodness-of-fit (*R*2) for each dataset exhibits a notable improvement when employing the high and low-frequency hierarchical learning method alongside the LSTM network. Additionally, the prediction accuracy (*P*) of the regression values demonstrates significant enhancement.

### 3.3. Highly Accurate Crack Width Modeling Based on Temperature Field Data

While the accuracy of the individual crack width models can be enhanced through the current LSTM framework, further improvements are necessary to achieve engineering significance. Currently, the temperature sensor data from the main tower have not been upgraded, as the monitoring system remains unchanged. This limitation hinders the development of temperature difference data along the wall thickness of the main tower, given that there are only four external temperature sensors (JW-T1 to JW-T4) installed on it. To address this issue, this study supplements the temperature field data of the bridge tower by constructing vertical temperature differences and analyzing the temperature disparities between the two towers. A total of four temperature difference groups, designated WC-1 to WC-4, have been created. Specifically, WC-1 represents the temperature difference between sensors JW-T1 and JW-T2, indicating the longitudinal temperature difference of the main tower upstream on the lodging side, while the remaining groups are developed using a similar methodology.

**Figure 11:**
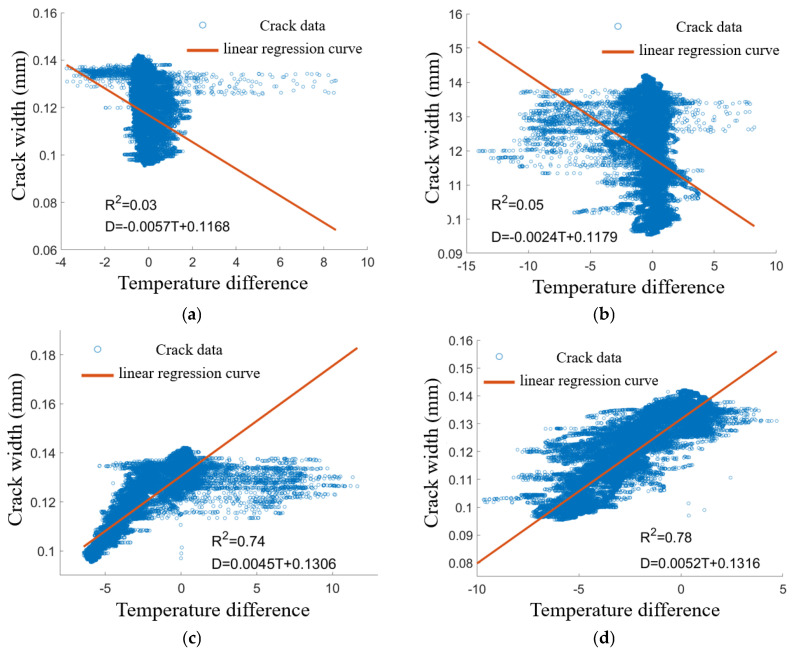
Correlation analysis of sensor LF-12-01 data with four temperature difference datasets: (**a**) LF-12-01 and WC-1. (**b**) LF-12-01 and WC-2. (**c**) LF-12-01 and WC-3. (**d**) LF-12-01 and WC-4.

Figure 11 illustrates the correlation analysis of the crack sensor LF-12-01 data with four sets of temperature difference data. It is evident that LF-12-01 exhibits a strong correlation with WC-3 and WC-4, with goodness-of-fit values for the linear regression equations of 0.74 and 0.78, respectively. In contrast, LF-12-01 shows a weak correlation with WC-1 and WC-2, suggesting that changes in crack width are not significantly associated with these temperature differences. Notably, most crack width values are clustered around the zero value of the temperature difference, with goodness-of-fit values for the linear regression equations of only 0.03 and 0.05, respectively. A similar correlation analysis was conducted for the remaining three cracks, yielding comparable conclusions. Specifically, LF-12-01 demonstrated weak correlations with WC-1 and WC-2, indicating a minimal relationship between changes in crack width and temperature differences, with the majority of crack width values again concentrated near the zero value. Consequently, after analyzing all four cracks, the longitudinal temperature difference data for the main tower will not be considered further. Instead, WC-3 and WC-4 will be utilized as supplementary data to construct the temperature field of the bridge’s main tower.

For the four datasets ranging from LF-12-01 to LF-12-04, the LSTM neural network model utilizing temperature field data is trained according to the established algorithmic process while simultaneously employing a high and low-frequency hierarchical learning method. The model’s input parameters are configured as follows: the minibatch size is set to 32, the initial learning rate is 0.0001, and the LSTM neural input layer consists of a total of 60 units. Additionally, the model incorporates two hidden layers, each containing 128 hidden units. Since three temperature features are utilized, the input size is adjusted to three. The computational accuracy of the LSTM model based on temperature field data is summarized in Table 4.

## 4. Crack Width Anomaly Perception

### 4.1. Uncertainty Analysis of LSTM Model Regression Values

**Figure 12:**
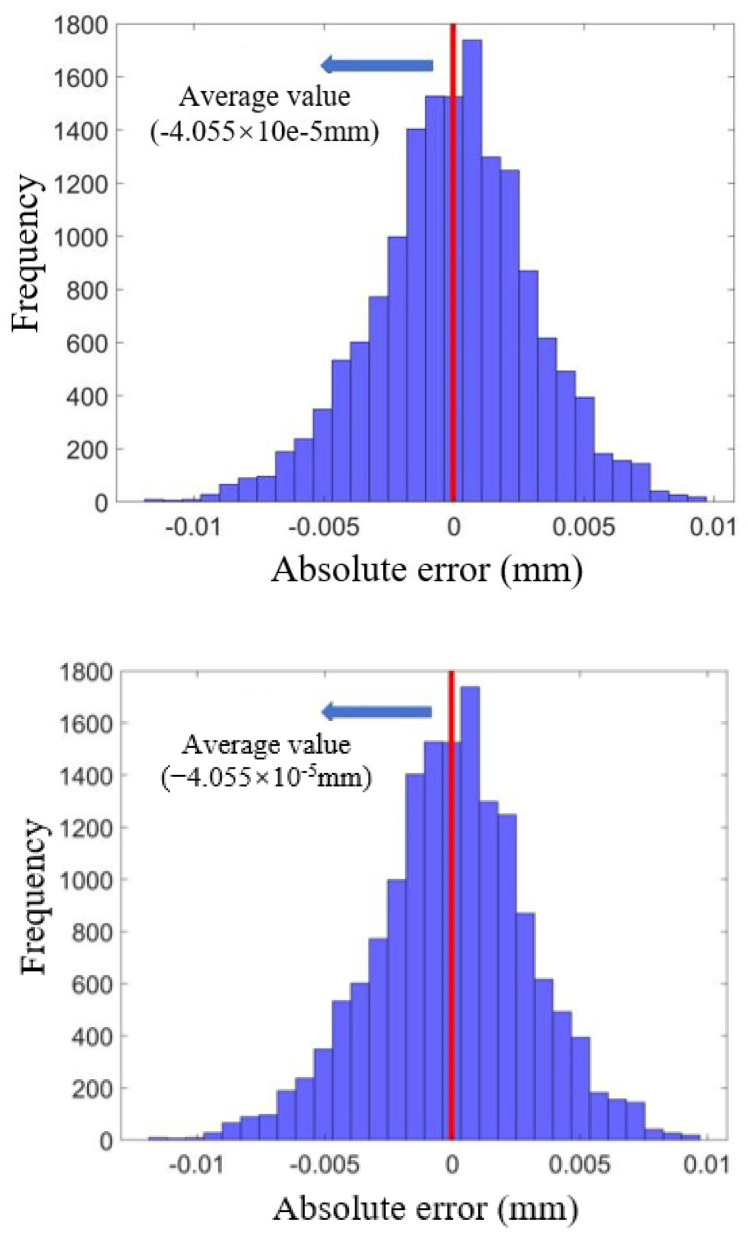
Histogram of error AE for stacked LSTMs.

**Figure 13:**
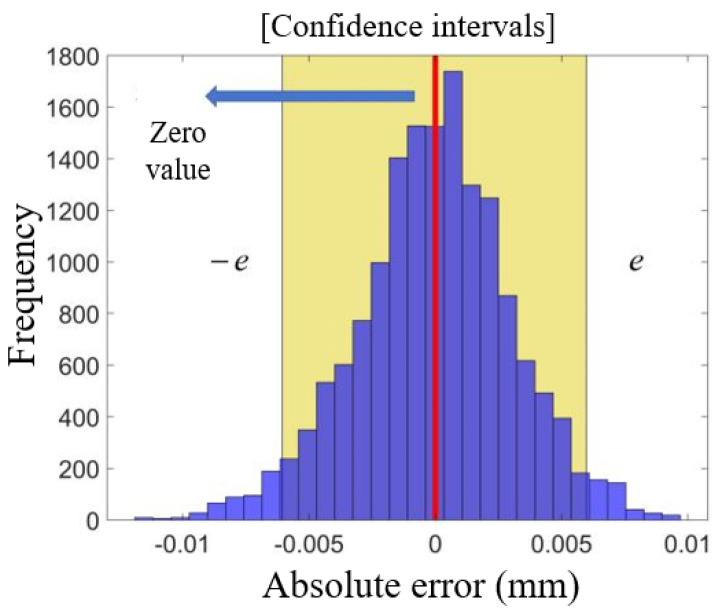
Confidence intervals with zero as a point of symmetry.

The histograms of the AE error values for the LSTM model corresponding to the crack sensor LF-12-01 test set are presented in Figure 12. The analysis reveals that the AE values of the test set exhibit a clear symmetric distribution, with the average AE value closely approximating zero. To ensure that the output results of the constructed LSTM network model can be directly applied in practice, an uncertainty interval centered around zero, ranging from ([−e, e]), is employed as a measure. A confidence level of 95% is established, referencing existing results in the field of bridge monitoring and early warning, as depicted in Figure 13.

As the confidence interval gradually increases and the confidence level ultimately reaches 95%, the resulting confidence interval is ([−0.006 mm, 0.006 mm]). Given the relatively small size of the crack itself, a confidence interval range of 0.012 mm may be excessive for certain practical applications, potentially affecting the sensitivity of anomaly detection and the perception of crack width. To achieve a more precise estimate of the anomalous crack width, it is essential to reduce the uncertainty of the model output without altering the magnitude of the regression value.

Typically, the probability distribution of model errors is symmetric, and according to the law of large numbers, the mean of a sufficiently large number of AE data points will converge to zero. By leveraging the high accuracy and robustness of the stacked LSTM model, the uncertainty of the model output values can be minimized using the sliding average method. This approach is applied to process both the measured crack width (*D*) and the regression value (*D’*), thereby reducing uncertainty. Notably, increasing the moving window length (*p*) results in decreased uncertainty. For instance, with a window length (*p*) of 100, the uncertainty interval is reduced to ([−0.0043 mm, 0.0043 mm]) at a 95% confidence level.

### 4.2. Numerical Sensing of Anomalous Crack Widths

The regression value (*D’*) obtained from the LSTM network model serves as a baseline, while (*D*) denotes the measured value of crack width influenced by temperature. Consequently, the Residual Absolute Deviation (RAD) between (*D*) and (*D’*) is used to represent the anomalous crack width value. The RAD is calculated using the following method:

(7) $$R_{AD}=D-D^{\prime }$$

**Figure 14:**
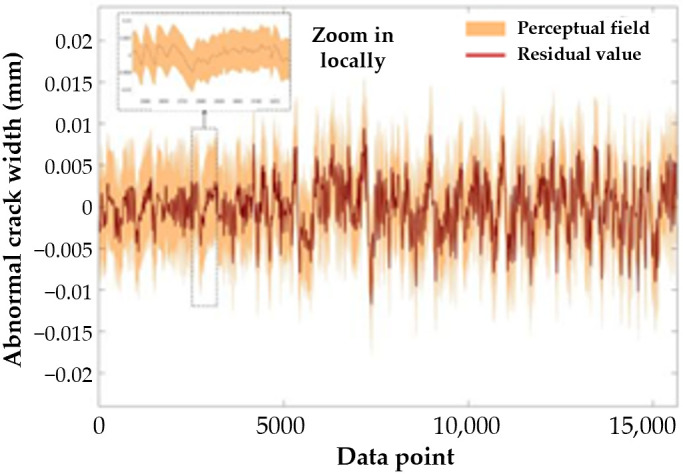
RAD and Perceptual Domain.

As illustrated in Figure 14, the Residual Absolute Deviation (RAD) calculated from the unprocessed raw data fluctuates around zero. In practical engineering applications, the potential range of anomalous crack width values should be considered as a sensing domain centered on the residual RAD curve, with a width of 0.012 mm (ranging from −0.006 mm to 0.006 mm). To accurately determine the specific anomalous crack width value using the residual RAD, it is essential to establish a definitive value. Consequently, defining the permissible error for the perception domain is crucial.

**Figure 15:**
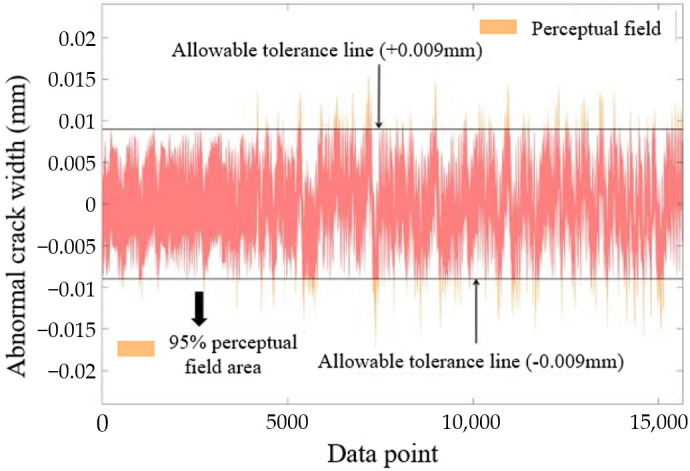
Perceptual domain and allowable error.

Given that the ideal value of the residual RAD should be zero, two permissible error lines symmetrical to this zero value are defined, as depicted in Figure 15. The values associated with these two symmetric allowable error lines, which encompass 95% of the area of the perceptual domain, are designated as the allowable error based on the 95% confidence level. For the RAD of the unprocessed raw crack data, the allowable error is calculated to be ±0.009 mm.

The allowable error lines establish a detection interval, with the residuals between these lines serving as the median value of the interval. Consequently, residual values (RAD) that fall within different intervals can be interpreted hierarchically, as referenced in Table 5. The raw data undergo processing through a sliding average, with the window length (*p*) set to 100 data points [24]. The RAD is then calculated from the processed values (*D’*) and (*D*). When the uncertainty is reduced to the range of ([−0.0043 mm, 0.0043 mm]), it is observed that the width of the sensing domain narrows to 0.0086 mm, resulting in a permissible error reduced to ±0.006 mm. As illustrated in Table 6, when the sensing sensitivity is enhanced to the order of 0.012 mm, the sensing frequency is adjusted to once every 500 min (equivalent to 100 data points).

It was further observed that variations in the sliding average window length influence both the monitoring frequency and the accuracy of the perceived anomalous crack width values. While the raw data can detect smaller anomalous crack widths with a larger sliding window length, this may compromise the timeliness of perception. According to the results presented in Table 7, when the sliding average window length reaches 1000 data points, the sensing accuracy for abnormal crack widths in the dataset improves to ±0.003 mm. Table 8 displays the sensing accuracy of each crack measurement point on the main tower of Huai’an Bridge under different sensing timeliness scenarios, indicating that satisfactory sensing sensitivity has been achieved for all crack measurement points on the bridge. It is worth noting that this study is limited in scope and has not been extended to validate other bridge types, but the concept of time-varying crack and temperature studies is similar, and thus serves as a valuable reference for analogous studies.

## 5. Conclusions

In this paper, we establish a mapping from complex temperature fields to the characteristics of crack temperature changes using a deep learning model. The main conclusions are as follows:

(1) A correlation analysis was conducted on the temperature and crack monitoring data for Huai’an Bridge over an 8-month period, which included data cleaning and wavelet packet decomposition. This analysis, involving four crack measurement points, revealed a strong relationship between crack width and temperature, with the highest goodness-of-fit (*R*2) reaching 0.9595. However, the performance of the univariate linear regression was inadequate, resulting in a prediction accuracy (*P*) of only 76.23%.
(2) For the numerical mapping regression of temperature-induced crack width, a stacked LSTM network model was established following parameter optimization. This approach significantly enhanced prediction accuracy and robustness compared to both the BP neural network (BPNN) and the univariate linear regression model. To further improve local learning effectiveness and model prediction accuracy, a layered learning method was proposed. The prediction accuracy after applying this layered learning approach reached 87.11%, surpassing the 79.58% achieved through direct use of the LSTM model.
(3) By supplementing the temperature difference data among the four temperature measurement points, the local representation of temperature field characteristics was enhanced. Consequently, a high-precision real-time crack width prediction model based on temperature field data was established, achieving a model regression goodness-of-fit (*R*2) of 0.9938 and a prediction accuracy (*P*) of 93.71%.
(4) Using the regression value from the stacked LSTM model with direct input data as the benchmark control value, the anomalous crack width can be represented as the residual difference between the measured value and the regression value. By establishing an allowable error, the numerical magnitude of the anomalous crack width for the main tower can be accurately determined. Analysis of the raw data from Huai’an Bridge reveals that the perceived sensitivity of the abnormal crack width at each measurement point is approximately ±0.012 mm. Furthermore, when the raw data are processed using the sliding average method, the maximum perceptual sensitivity at each measurement point can be increased by approximately ±0.0022 mm.

Although this study has established a crack LSTM prediction model applied to the Huai’an Bridge, subsequent studies should consider assessing the transferability to other bridges or datasets. In addition, research into Gated Recurrent Units (GRUs), transformer-based models, and their optimization represents avenues for future exploration. Furthermore, extensive empirical validation is necessary to enhance real-time crack monitoring and reduce maintenance costs.

## Figures and Tables

**Table 1:** Neural network data input–output supervised learning strategies for datasets over time.

Point in Time	Input Data	Output Data	Input Data	Output Data
30	T_1(1)_, T_1(2)_, …, T_1(59)_, T_1(60)_	*D*_1(30)_ or *D*_2(30)_	T_2(1)_, T_2(2)_, …, T_2(59)_, T_2(60)_	*D*_2(30)_ or *D*_4(30)_
31	T_1(2)_, T_1(3)_, …, T_1(60)_, T_1(61)_	*D*_1(31)_ or *D*_2(31)_	T_2(2)_, T_2(3)_, …, T_2(60)_, T_2(61)_	*D*_3(31)_ or *D*_4(31)_
32	T_1(3)_, T_1(4)_, …, T_1(61)_, T_1(62)_	*D*_1(32)_ or *D*_2(32)_	T_2(3)_, T_2(4)_, …, T_2(61)_, T_2(62)_	*D*_3(32)_ or *D*_4(32)_
…	…	…	…	…

**Table 2:** Comparative analysis of the effects of different learning methods.

Learning Methods	Low-Frequency Section (*ρ_R_*)	High-Frequency Section (*ρr*)	Test Set Regression Value Prediction Accuracy (*P*)	Test Set Regression Value Goodness of Fit (*R*2)
Direct method of importing	99.13%	33.96%	79.58%	0.9809
High-frequency and low-frequency layering	99.57%	59.51%	87.11%	0.9747

**Table 3:** Computational accuracy of LSTM model for each sensor test set data.

Sensors	One-Dimensional Linear Regression (*R*2)	LSTM Network Regression (*R*2)	One-Way Linear Regression *p*-Value	LSTM Network Regression *p*-Value
LF-12-01	0.9518	0.9747	76.78%	87.55%
LF-12-02	0.9595	0.9698	69.63%	86.55%
LF-12-03	0.9363	0.9639	68.86%	86.70%
LF-12-04	0.9292	0.9689	65.39%	85.42%

**Table 4:** Computational accuracy of LSTM model based on temperature field.

Sensors	One-Dimensional Linear Regression (*R*2)	LSTM Network Regression (*R*2)	One-Way Linear Regression *p*-Value	LSTM Network Regression *p*-Value
LF-12-01	0.9518	0.9934	76.78%	93.51%
LF-12-02	0.9595	0.9932	69.63%	93.61%
LF-12-03	0.9363	0.9922	68.86%	93.71%
LF-12-04	0.9292	0.9938	65.39%	93.10%

**Table 5:** Perceived sensitivity of anomalous crack width in the main tower using residual RAD (range in mm).

The residual RAD falls into the range	…	−0.045 to −0.027	−0.027 to −0.009	−0.009 to −0.009	0.009~0.027	0.027~0.045	…
Abnormal crack width perception	…	−0.036	−0.018	0	0.018	0.036	…

**Table 6:** Perceived sensitivity of anomalous crack widths in the main tower using residual RAD (after sliding average of *p* = 100).

The residual RAD falls into the range	…	−0.030 to −0.018	−0.018 to −0.006	−0.006 to −0.006	0.006~0.018	0.018~0.030	…
Abnormal crack width perception	…	−0.024	−0.012	0	0.012	0.024	…

**Table 7:** Effect of processing window length on perceived timeliness and perceived sensitivity of anomalous crack width values.

Sliding Average Processing Window Length *p* (Number of Data Points)	Perception Time Limit (min)	95% Confidence Interval (mm)	Perceived Sensitivity of Abnormal Crack Width (mm)
0 (original data)	5	[−0.0060, 0.0060]	±0.009
50	250	[−0.0049, 0.0049]	±0.0075
100	500	[−0.0043, 0.0043]	±0.006
250	1250	[−0.0030, 0.0030]	±0.005
500	2500	[−0.0018, 0.0018]	±0.003
1000	5000	[−0.0013, 0.0013]	±0.002

**Table 8:** Perceived sensitivity analysis of crack measurement points of Huai’an Bridge main tower.

Measurement Point	Perception Time Limit 5 Min	Average of 5 Min of Perceptual Aging	Perception Time Limit 500 Min	Average of 500 Min of Perceptual aging	Perception Time Limit 5000 Min	Average of 5000 Min of Perceptual Aging
LF-12-01	±0.009 mm	±0.012 mm	±0.006 mm	±0.0071 mm	±0.002 mm	±0.0022 mm
LF-12-02	±0.012 mm		±0.0075 mm		±0.0022 mm	
LF-12-03	±0.012 mm		±0.007 mm		±0.0021 mm	
LF-12-04	±0.014 mm		±0.008 mm		±0.0024 mm	

## Data Availability

The raw data supporting the conclusions of this article will be made available by the authors on request.
